# Chart Review Is Dead; Long Live Chart Review: How Artificial Intelligence Will Make Human Review of Medical Records Obsolete, One Day

**DOI:** 10.1089/pop.2023.0227

**Published:** 2023-11-24

**Authors:** Kevin Agatstein

**Affiliations:** KAID Health, Boston, Massachusetts, USA.

Many clinical and administrative workflows, including billing and coding, quality measurement, medical management, care management, prior authorization, and patient risk assessment, require various forms of chart review. Chart review is when medical records, some years old and some newly created, are analyzed to extract one or more clinical facts. Such summarization could involve identifying a coding error or omission, making an outcome measure reportable, extracting laboratory values from free text to appropriately apply a clinical guideline, or identifying a clinical trial candidate.

The cost of this process is staggering. For example, Johns Hopkins Hospital in Baltimore, Maryland spends over $5 million on quality reporting alone, with collecting and validating electronic medical record data being a major driver of that spending.^1^ The burden of chart review is not limited to support staff. Overhage and McCallie report that nonsurgical physicians spend more than 5 minutes per care encounter reviewing medical charts.^2^

Fortunately, with artificial intelligence (AI), specifically natural language processing (NLP), much of the burden of reviewing charts is now automatable. Or is it? NLP in this context is a computer's ability to interpret human language (eg, unstructured text in a medical note) and express the author's intent as structured data such as diagnoses or procedure codes. Such AI-led chart review is at the same time inferior to, superior to, and fundamentally different from having humans do chart reviews.

## Human Intelligence Still Trumps AI for Complex Chart Reviews

For many clinical decisions, AI is more reliable than health care professionals. For example, AI can often detect barely visible lesions in an imaging study or predict cancer recurrence from a tumor genome better than well-trained physicians. However, when it comes to extracting relevant information from a clinical chart and summarizing the patient's data, clinicians do many things better than computers.

### Humans can better understand context

Human reviewers have an innate ability to understand what is written in a medical chart. For example, a human reviewer will almost certainly correctly deduce from the sentence “General: No acute distress, Chest: CTA, ABD: bowel sounds normal,” that CTA means “clear to auscultation,” even if nothing else is written on the page. In other contexts, CTA could stand for CT-angiogram, a radiological study of the chest area.

### Humans can better integrate disparate data

When reading a section of a chart, other pieces of clinical data can aid a human in interpretation. Consider the sentence, “PAP normal.” Knowing the patient to be male, a human would deduce that this likely refers to a pulmonary arterial pressure rather than the more common use of PAP referring to cervical cancer screening. Here, the patient's gender, not specifically noted in the sentence, changes the interpretation of the result. Integration of disparate data elements to create a uniform understanding is a natural human trait, but very challenging for AI.

### Humans can better detect noise

Medical records are messy in every sense of the word. Handwriting is poor. Forms are complicated. Spelling and grammar errors are pervasive, pages are missing, and data elements are contradictory. For example, consider the note fragment “PD-11 (55%).” Most oncologists would recognize that the first “1” should be an “L” for PD-L1, even if the document scanner did not. That example is just the tip of the tip of the iceberg.

### Humans can see subtleties, nuances, and what is not written

There is more to medical records than the specific text or numeric values. For example, consider a pediatrician's note reading, “As the patient is homeschooled with irregular access to care, the medication regimen selected was optimized to aid in compliance.” Although nothing in this note says this patient is not adhering to clinician advice, some may interpret the sentence to suggest a compliance issue.

### Humans can dig deeper

Unlike most implementations of AI, clinicians can say, “I don't have all I need here.” Humans are more likely than AI to say a page is missing or a required laboratory value is not in the chart. For example, a mention of a referral to the emergency department for suicidal ideation with no other mentions of behavioral health issues creates a sense to the reviewer that relatively important pieces of data are missing from the record.

So, people are still needed for some form of chart review. Fortunately, there is still a meaningful role for AI.

## For Simple Tasks, AI Outperforms the Clinician

Although medical, nursing, and billing and coding training programs do not need to close their doors just yet, there are some elements of chart review that computers are demonstrably better at. This is especially true for extraction of relatively routine data elements (eg, presence or absence of a disease, laboratory value, testing) where little integration of disparate data is required. Here, AI outperforms human reviewers in several ways.

### AI is more consistent at finding things

As would be expected, computers outperform clinicians in repetitive tasks. For example, Suh et al. found that AI was superior to physicians at extracting a predefined set of key anesthesia risk factors from the medical charts of preoperative patients.^3^ Finding METS scores or depressed renal function in a medical record is relatively easy. Furthermore, for a computer, it is just as easy to do this on the thousandth chart of the day as the first. The same is not true for a flesh-and-blood clinician.

### AI lets fewer things “fall through the cracks”

AI simply has better memory than humans. For example, in looking for patients with a coagulopathy, a human would need to recall the hundreds of different medical conditions, hereditary and acquired, that impede blood clotting. This is not an easy task. Looking for hundreds of different diseases at one time is simply easier for computers. Both human and computer would likely not miss tagging hemophilia or von Willebrand disease in a chart. The same may not be true for plasminogen deficiency or alpha 2-antiplasmin deficiency.

### AI can be less biased, at least in some ways

Tasks that involve human judgement are at risk for significant bias. This holds true in chart review and summarization. For example, if a reviewer has prior belief, conscious or not, that a patient will have or not have a specific finding in their chart, she may be more likely to confirm her suspicion. For example, Seng et al. showed that AI can identify patients who have failed to ween off opioids after orthopedic procedures.^4^ Although there are risks of biases in the application of AI, in this example the reviewer, the AI, did not have any preconceived impression on the likelihood of long-term pain control in the chart. It did not look harder, or less hard, on one chart versus another. Some human reviewers may not be as disciplined in their approach.

### AI is profoundly cheaper

Finally, computer processing time and data storage are trivial costs compared with high-trained clinical labor, and even unlicensed support staff. A comprehensive medical chart review could cost in excess of $50 or more for certain diseases, whereas AI analysis of records, even without scale, is a fraction of this expense.

So if human versus AI is Coke versus Pepsi, who wins? Well, hard to say, as it is probably closer to apples versus oranges.

## AI-Based Chart Review Is Fundamentally Different than Human Review

Rather than simply comparing the performance of AI versus humans in reviewing charts, it is useful to appreciate how the approaches are fundamentally different. Specifically, the scalability and cost-efficiency of AI-supported chart reviews change the very nature of chart review in 3 fundamental ways.

### With AI, everything in a chart can be found at once

Today, a quality team may look for HEDIS gaps in a chart, a care management team may look for social determinants of health for the same patient, and a coding team may look for evidence of a comorbid condition. Cardiology may be looking for results of past stress tests, whereas nephrology looks for the estimated glomerular filtration rate values faxed in from an external laboratory. Tomorrow, a new patient safety initiative will have users finding all lung nodules that have not been reassessed recently. The list is endless. Rather than having each group constantly reopen the record, AI allows each of the aforementioned to be answered on demand, and new inquiries added in real time.

### With AI, charts can be reviewed repeatedly

The cost of reviewing medical records means it must be done judiciously, both in terms of what is looked for and the frequency of review. AI can all but eliminate that constraint. Each new piece of data added to the chart can be automatically analyzed. Rather than generating periodic snapshots of the patient, AI allows patient analytics to keep pace with the patient's clinical progression.

### With AI, findings can be automatically coded and organized

AI not only finds relevant insights in the medical record but also organizes them. For example, there may be hundreds of different ways “arrhythmia” is characterized in a medical note (eg, “A-fib,” “flutter,” “SVT”). In some cases, each of these means different things. In other cases, the aggregate concept is what is important. Because AI can identify clinical concepts of interest and assign one or more medical codes to them, analysis can be done at any aggregation level.

The aforementioned are not just examples of how AI is better than humans at the chart review task today, rather they describe entirely different uses of the process.

## The Best Approach, “the Genius of the “and” Versus the Tyranny of the “or””

Applying AI in complex process automation is seldom as simple as replacing traditional workflow with a new technology. This holds true for chart review. However, armed with an understanding of the strengths and limitations of each approach, novel solutions become clear.

To harness the power of AI in chart review, it is necessary to employ a 2-step strategy. First, let the AI summarize the chart as best as possible, extracting key insights and findings. In some cases, this may be enough. Others, where the limitations of AI may introduce intolerable data quality issues, a human reviewer can then review the findings, remove ambiguities, resolve unclear or contradictory data elements, address legibility issues, and dig deeper into issues of importance, all as needed. Fortunately, technology in general, including AI, can even streamline this manual review process.

When a human is reviewing a chart, AI can surface the most relevant examples of a specific finding being sought and suppress less clear ones. For example, a reviewer looking for evidence of heart failure will want to see the sentence “Hx chronic CHF, dx 2019.” She will not have an interest in the sentence “Patients with heart failure should avoid NSAIDs.” While AI will find the reference to heart failure in both these examples, it can also quantify the degree to which the 2 actually suggest the patient has the disease, high in the first sentence, low in the second. Such a prioritization of findings greatly simplifies human review.

Finally, the same errors and uncertainties rarely appear once in a set of charts. Sometimes, the same unclear sentence is cut and pasted a hundred times. For example, a set of notes may have hundreds of statements such as “referred to BH.” Whereas AI might suggest this means referred for behavioral health evaluation, in this example it actually meant “referred to Bonnie Haig* (the audiologist at the clinic).” An ability to bulk correct AI's interpretation of the record analysis greatly improves overall process efficiency.

## Computers and Humans Working Hand in Hand, At Least for Now

Skilled reviewers, empowered by AI and supporting workflow technology, can make chart review less expensive, more accurate, and more productive. Such computer-assisted chart review will be needed to meet the increasing demands of health care purchasers and regulators, as well as ever more sophisticated clinical guidelines.

The promise of AI for this task is just starting to be realized. In the long term, many believe AI's performance will improve so much that it will be equivalent to a human reviewer in all meaningful respects. At the same time, HAL9000 will care for its crew, the Tesla will safely auto drive through end-of-day pickup at an elementary school, and the chatbot will replace the ED doc to rule out a myocardial infarction. Until then, effectively piecing together the complementary strengths of AI and human reviewers will unlock massive value for patients and financial windfalls for those health care organizations that best employ the approach.

## Notes

^*^
Bonnie Haig is a fictitious name used purely for illustrative reasons. Any resemblance to a real person is purely coincidental.
